# Three new species of western California springsnails previously confused with *Pyrgulopsis stearnsiana* (Caenogastropoda, Hydrobiidae)

**DOI:** 10.3897/zookeys.601.9040

**Published:** 2016-06-29

**Authors:** Robert Hershler, Hsiu-Ping Liu, Caitlin Babbitt, Michael G. Kellogg, Jeanette K. Howard

**Affiliations:** 1 Department of Invertebrate Zoology, Smithsonian Institution, Washington, D.C. 20013-7012, USA; 2 Department of Biology, Metropolitan State University of Denver, Denver, CO 80217, USA; 3 San Francisco Public Utilities Commission, 525 Golden Gate Avenue, San Francisco, CA, 94102, USA; 4 The Nature Conservancy, 201 Mission Street, San Francisco, CA 94105, USA

**Keywords:** Gastropoda, United States, freshwater, taxonomy, conservation

## Abstract

We describe three new, allopatric species of springsnails (genus *Pyrgulopsis*) from western California (*Pyrgulopsis lindae*, *Pyrgulopsis ojaiensis*, *Pyrgulopsis torrida*) that were previously identified as *Pyrgulopsis stearnsiana*. The new species are differentiated from *Pyrgulopsis stearnsiana* and each other both by mtCOI sequences (3.9-9.9%) and details of penial morphology. We also provide a phylogeny with increased sampling which confirms a previous finding that *Pyrgulopsis stearnsiana* *sensu stricto* is paraphyletic relative to two other California species (*Pyrgulopsis diablensis*, *Pyrgulopsis giulianii*). Our molecular and morphological evidence suggests that *Pyrgulopsis stearnsiana* paraphyly is an artifact of conservative taxonomy, however additional studies utilizing rapidly evolving genetic markers will be needed to confidently tease apart the cryptic diversity in this widely ranging springsnail. The new species described herein are narrowly distributed and vulnerable to anthropogenic stressors. The single known population of *Pyrgulopsis torrida* may have become extirpated between 2000 and 2015.

## Introduction

*Pyrgulopsis* Call & Pilsbry, 1886 is a large genus (139 species; Hershler et al. 2014a) of hydrobiid gastropods (commonly known as springsnails) that is distributed in springs and other groundwater-dependent habitats throughout much of western North America from the Missouri River headwaters and Rio Grande Basin to the Pacific margin, and from the lower Columbia River to the Rio Nazas-Rio Aguanaval basin (Hershler et al. 2014b). Most of these tiny snails have very narrow geographic ranges consisting of a single spring, spring system or local watershed (Hershler et al. 2014b). Molecular studies have shown that several of the more widely ranging members of this genus are composites of divergent lineages. This is the third in a series of papers that revises the taxonomy of these species (Hershler et al. 2013, Hershler et al. 2014a).

*Pyrgulopsis stearnsiana* (Pilsbry, 1899) (= *Paludestrina stearnsiana* Pilsbry, 1899) was described for small (2.6 mm), narrowly umbilicate, ovate-conic shells from “near Oakland” (type locality), two additional localities in the San Francisco Bay area, and “Tuolumne County” (located along the western flank of the Sierra Nevada). Taylor (1981) subsequently expanded the range of *Pyrgulopsis stearnsiana* to include a large portion of the central and southern California coast. Hershler (1994) provided a detailed description and illustrations of *Pyrgulopsis stearnsiana* from Palo Seco Creek in Oakland and emended the diagnosis by adding details of penial morphology. A recent phylogenetic analysis resolved mtCOI sequences from six *Pyrgulopsis stearnsiana* populations into four evolutionarily distinct, allopatric lineages (Hershler and Liu 2010). One of the lineages was composed of specimens from just north of Oakland (Wildcat Canyon, San Pablo Creek drainage) and two localities in central California coastal drainages; two regional congeners (*Pyrgulopsis diablensis* Hershler, 1995; *Pyrgulopsis giulianii* Hershler and Pratt, 1990) were also nested within this clade (also see Liu et al. 2003). The other lineages were single populations from the Sierra Nevada foothills, and near the southern edge of the species’ range. Here we detail previously unreported morphological differences among these lineages and describe three of them as new species based on our combined (molecular and morphological) evidence. We also provide a molecular phylogeny with additional sampling in the *Pyrgulopsis stearnsiana* clade and discuss the taxonomic status of this group.

## Methods

**Figure 1.**
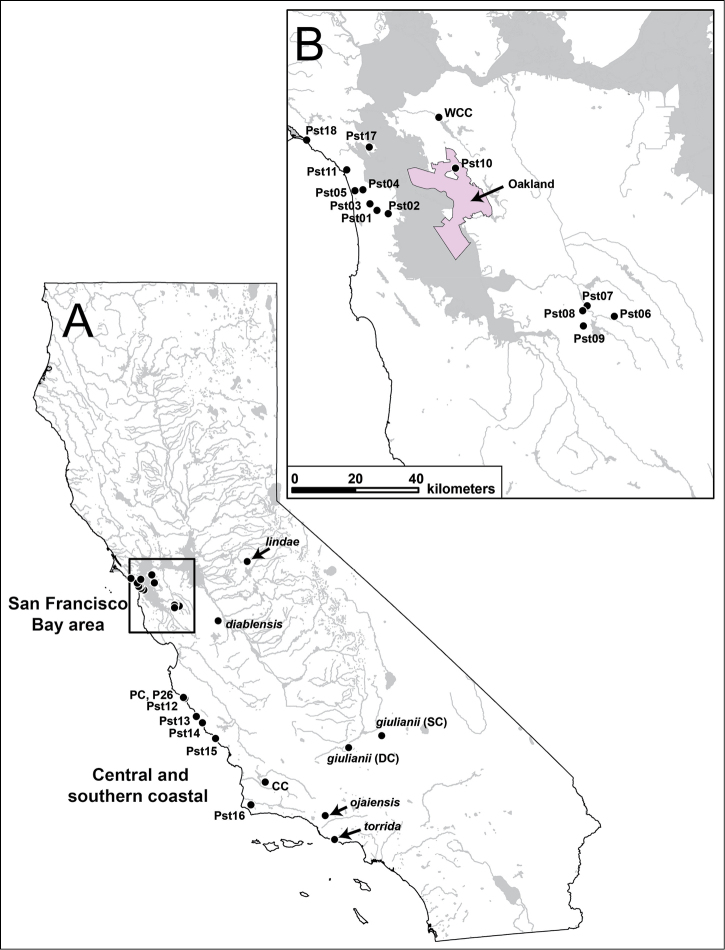
Map of California (**A**) and San Francisco Bay area (**B**, area occupied by rectangle in **A**) showing the collection localities for samples of *Pyrgulopsis stearnsiana* (and closely related *Pyrgulopsis diablensis* and *Pyrgulopsis giulianii*) and the three new species (highlighted by arrows) that were used in the molecular analysis. Specimen codes are from Suppl. material 1.

During 2014 and 2015 we sampled 18 additional *Pyrgulopsis stearnsiana* populations in the San Francisco Bay area (including one in Oakland) and central and southern California coastal drainages (Fig. 1, Pst1-Pst18). Some of these are new localities while the others are previously known records documented in museum collections and/or an unpublished monograph on the genus *Fontelicella* Gregg & Taylor, 1965 (a junior synonym of *Pyrgulopsis*) by Wendell Gregg and Dwight Taylor. Specimens were collected by hand or with a small sieve and preserved in 90% (non-denatured) ethanol in the field for mtDNA analysis. Portions of the larger samples were anaesthetized with menthol crystals (for 13 hours), fixed in dilute formalin (10% of stock solution), and preserved in 70% ethanol for subsequent anatomical study. GPS coordinates were taken at each (snail-positive) site using a hand-held unit (Garmin Oregon 450t).

Genomic DNA was extracted from entire snails (3-6 specimens per sample) using a CTAB protocol (Bucklin 1992); each specimen was analyzed for mtDNA separately. LCO1490 and HCO2198 (Folmer et al. 1994) were used to amplify a 710 base pair (bp) fragment of cytochrome *c* oxidase subunit I (COI), and ND43F and RND592F (Liu et al. 2003) were used to amplify a 550 bp fragment of NADH dehydrogenase subunit I (NDI). Amplification conditions and sequencing of amplified polymerase chain reaction product followed Liu et al. (2003). Sequences were determined for both strands and then edited and aligned using Sequencher™ version 5.0.1. The 76 newly sequenced specimens (69 COI and 72 NDI sequences) were analyzed together with previously published sequences of *Pyrgulopsis stearnsiana* (Hershler et al. 2003, Liu et al. 2003, Hershler and Liu 2010) and 11 congeners from California and southwestern Nevada; the collecting localities for the *Pyrgulopsis stearnsiana*, *Pyrgulopsis diablensis* and *Pyrgulopsis giulianii* samples are shown in Figure 1. The type species of the eastern North American genus *Marstonia* (a close relative of *Pyrgulopsis*; Hershler et al. 2003) was used to root the resulting trees. One example of each haplotype detected in a given sample was used in our analyses. Sample information for the sequences that were included in our analysis is detailed in Suppl. material 1.

We analyzed the COI and NDI datasets both separately and combined. MrModeltest 2.3 (Nylander 2004) was used to obtain an appropriate substitution model (using the Akaike Information Criterion) and parameter values for this analysis. Phylogenetic relationships were inferred by Bayesian analysis using MrBayes 3.1.2 (Huelsenbeck and Ronquist 2001). Metropolis-coupled Markov chain Monte Carlo simulations were run with four chains (using the model selected through MrModeltest) for 5,000,000 generations, and Markov chains were sampled at intervals of 10 generations to obtain 500,000 sample points. We used the default settings for the priors on topologies and the GTR + I + G model parameters selected by MrModeltest as the best fit model. At the end of the analysis, the average standard deviation of split frequencies was less than 0.01 and the Potential Scale Reduction Factor (PSRF) was 1, indicating that the runs had reached convergence. The sampled trees with branch lengths were used to generate a 50% majority rule consensus tree with the first 25% of the samples removed to ensure that the chain sampled a stationary portion. Genetic distances (maximum composite likelihood) within and between species/lineages were calculated using MEGA6 (Tamura et al. 2013), with standard errors estimated by 1,000 bootstrap replications with pairwise deletion of missing data.

The material collected during the course of this study was deposited in the National Museum of Natural History
(USNM) collection. Asterisked lots are vouchers for the new mtDNA sequences reported herein. Other relevant material from the USNM, Academy of Natural Sciences of Philadelphia
(ANSP), Bell Museum of Natural History
(BellMNH), and Santa Barbara Museum of Natural History
(SBMNH) was also examined during the course of this study. Specimens of *Pyrgulopsis stearnsiana* *sensu stricto* that were examined during the course of this study are listed in Suppl. material 2. Large adults were used for shell measurements. The total number of shell whorls was counted (WH) for each specimen; and the height and width of the entire shell (SH, SW), body whorl (HBW, WBW), and aperture (AH, AW) were measured from camera lucida outline drawings (Hershler 1989). Three ratios were generated from the raw data (SW/SH, HBW/SH, AH/SH). Descriptive statistics were generated using Systat for Windows 11.00.01 (SSI 2004). Other methods of morphological study were routine (Hershler 1994, Hershler 1998); descriptive penial terminology is from Taylor (1987) and Hershler (1994, 1998). Inasmuch as we have limited material for the new species, we have only provided brief taxonomic descriptions that are focused on diagnostic aspects of morphology.

## Results

**Figure 2.**
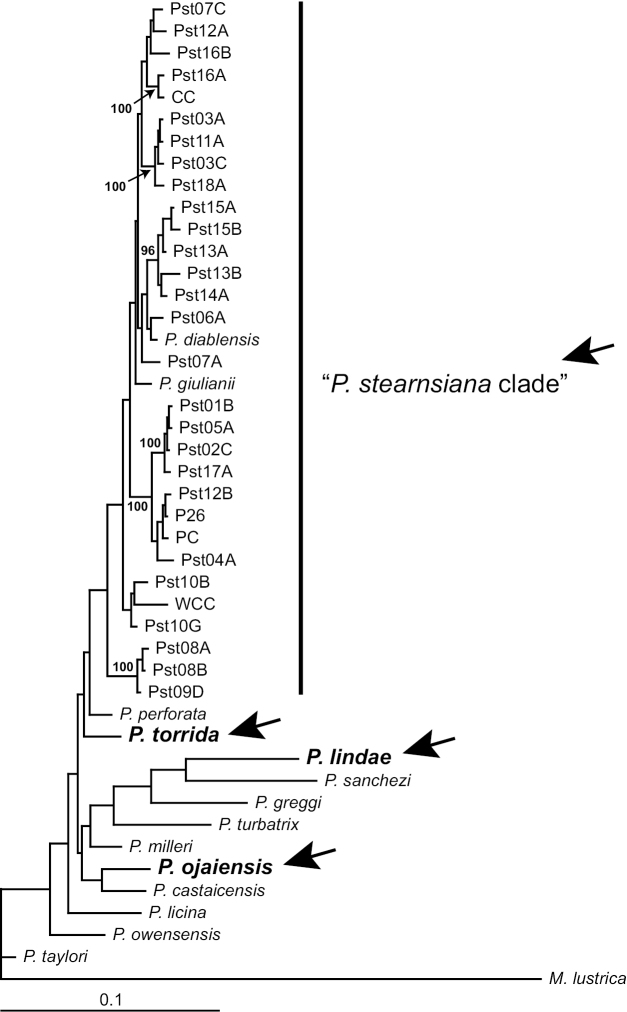
Bayesian tree based on the COI dataset. The four lineages of *Pyrgulopsis stearnsiana* *sensu lato* are identified by arrows. Posterior probabilities for nodes are shown when >95%. Specimen codes are from Suppl. material 1.

**Figure 3.**
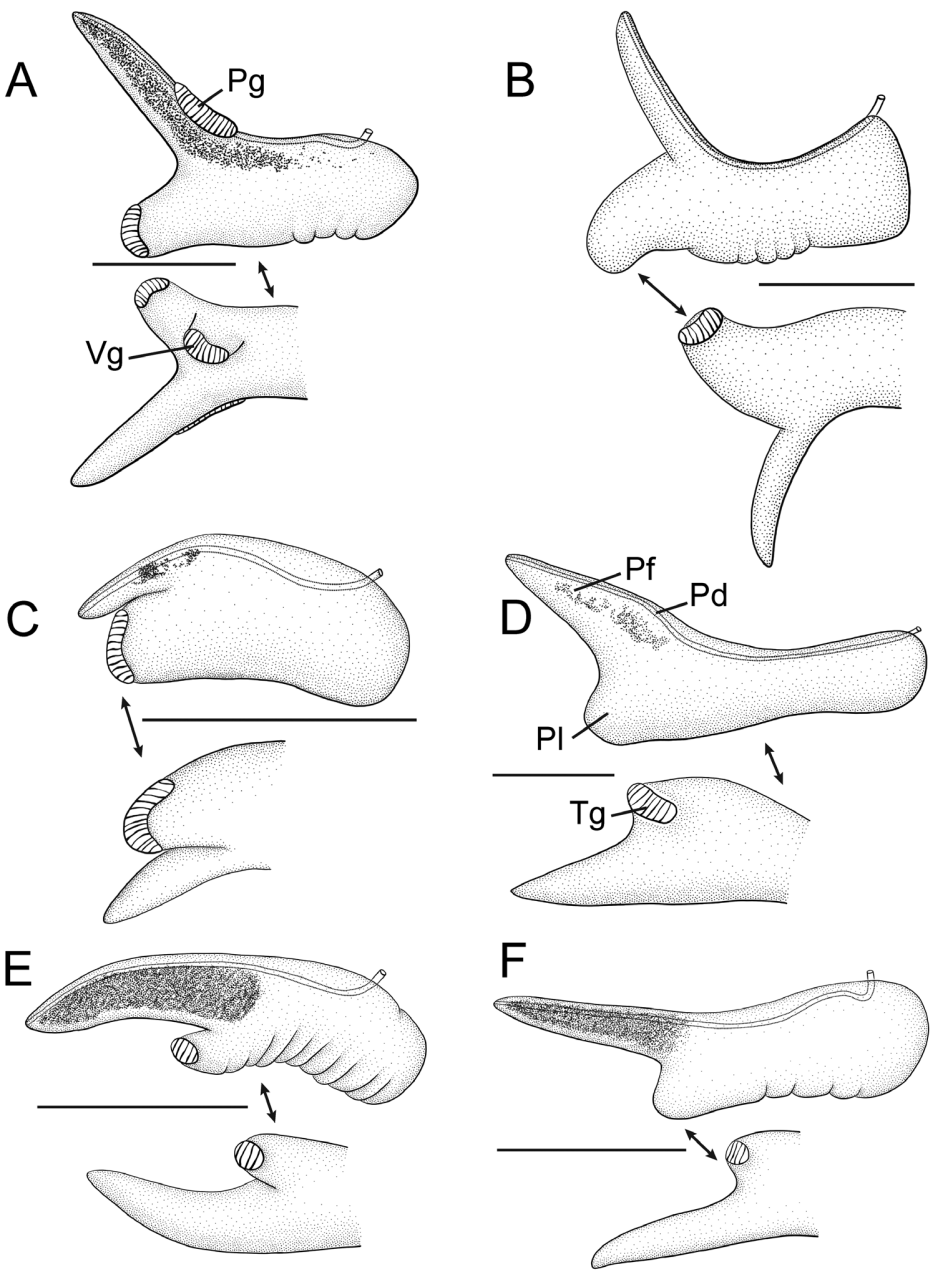
Penes (dorsal, ventral surfaces). **A** *Pyrgulopsis lindae* sp. n., USNM 1257409 **B** *Pyrgulopsis ojaiensis* sp. n., SBMNH 460496 **C** *Pyrgulopsis torrida* sp. n. USNM 1120443 **D, E, F** *Pyrgulopsis stearnsiana* USNM 1297168, USNM 1252041, USNM 905251, respectively. Scale bars: 250 µm. Pd penial duct
Pf penial filament
Pg penial gland
Pl penial lobe
Tg terminal gland
Vg ventral gland.

The Bayesian analysis of the COI dataset (Fig. 2) resolved specimens of *Pyrgulopsis stearnsiana* *sensu lato* into four distinct, allopatric lineages. These four lineages were also delineated in the otherwise poorly resolved NDI and combined (COI + NDI) Bayesian trees (not shown). The sister relationships of the four lineages of *Pyrgulopsis stearnsiana* *sensu lato* were not well supported. One of the lineages (referred to herein as the “*Pyrgulopsis stearnsiana* clade”) contained the newly sequenced specimens and other snails conforming to *Pyrgulopsis stearnsiana* as currently diagnosed—i.e., having a narrowly umbilicate and ovate-conic shell with medium convex whorls, and an elongate penial filament and very small penial lobe with a single gland along its distal edge (Hershler 1994). *Pyrgulopsis diablensis* and *Pyrgulopsis giulianii* were nested in this marginally supported (93% posterior probability) clade as in the previous published analyses. The other three lineages (consisting of single populations) are substantially divergent genetically, differing from *Pyrgulopsis stearnsiana* and each other by 3.9–9.9% for COI (Table 1); and are further differentiated by penial morphology (Fig. 3). (Note that we sequenced NDI for one of these lineages, described as *Pyrgulopsis ojaiensis* below, which differed from *Pyrgulopsis stearnsiana* *sensu stricto* by 6.0 +/- 1.0% for this marker; Table 2.) Our findings that these lineages are both genetically divergent and morphologically diagnosable suggests that they are distinct species, which we describe below.

**Table 1.** Per cent COI sequence divergence among *Pyrgulopsis* species included in the phylogenetic analyses. Values are mean +/- standard deviation.

	*stearnsiana*	*diablensis*	*giulianii*	*lindae*	*ojaiensis*	*torrida*
*stearnsiana*	2.0 +/- 0.3					
*diablensis*	1.5 +/- 0.3	-				
*giulianii*	1.9 +/- 0.4	1.1 +/- 0.4	-			
*lindae*	9.6 +/- 1.2	10.2 +/- 1.3	9.7 +/- 1.3	-		
*ojaiensis*	5.4 +/- 0.8	4.8 +/- 0.9	5.6 +/- 0.9	9.9 +/- 1.2	-	
*torrida*	3.9 +/- 0.7	3.5 +/- 0.8	3.5 +/- 0.8	9.4 +/- 1.2	4.8 +/- 0.9	-
other species	3.5–9.4	2.6–9.1	3.1–9.4	9.0–10.8	3.8–11.7	2.8–9.2

**Table 2.** Per cent NDI sequence divergence among *Pyrgulopsis* species included in the molecular phylogenetic analyses. Data are not available for *Pyrgulopsis lindae* and *Pyrgulopsis torrida*. Values are mean +/- standard deviation.

	*stearnsiana*	*diablensis*	*giulianii*	*ojaiensis*
*stearnsiana*	2.4 +/- 0.4			
*diablensis*	2.0 +/- 0.4	-		
*giulianii*	2.3 +/- 0.4	1.5 +/- 0.5	0.8 +/- 0.4	
*ojaiensis*	6.0 +/- 1.0	6.0 +/- 1.1	6.0 +/- 1.1	-
other species	5.2–10.4	5.1–10.5	4.6–10.5	5.4–11.3

### Systematic descriptions Family Hydrobiidae Subfamily Nymphophilinae

#### Genus *Pyrgulopsis* Call & Pilsbry, 1886

The three new species are assignable to *Pyrgulopsis* based on morphology, e.g., presence of a single seminal receptacle, diffuse mantle pigmentation, superficial position of the bursa copulatrix and its duct on the albumen gland (Liu and Hershler 2005); and molecular phylogenetic evidence.

##### Pyrgulopsis lindae

Taxon classificationAnimaliaLittorinimorphaHydrobiidae

Hershler, Liu, Babbitt, Kellogg & Howard sp. n.

http://zoobank.org/2C71096A-39EE-4808-AAB9-EEE6D6787D92

Figs 3A , 4

Pyrgulopsis stearnsiana .—Hershler and Liu 2010 (in part).

###### Types.

Holotype, USNM 905258, San Domingo Creek, 3.8 km up flow from Dogtown along San Domingo Road, Calaveras County, California, 38.14122°N, 120.53920°W, 6/26/2000, R. Hershler. Paratypes, *USNM 1254709 (one dry shell and six alcohol-preserved specimens), from same lot.

###### Referred material.

California. *Calaveras County*: ANSP 158719, Santo Domingo (probably San Domingo) Creek Valley, N (north) of Murphys, no coordinates available, 9/11/1929. *Tuolumne County*: BellMNH 20821, Salvada Gulch (37.87062°N, 120.41987°W), 11/9/1966.

###### Diagnosis.

A medium-sized congener (maximum shell height, 3.3 mm) having an ovate-conic shell. Distinguished from other regional species in having a penial gland along the outer edge of the filament. Further differentiated from *Pyrgulopsis stearnsiana* in having a ventral gland on the penis, and a larger penial lobe and terminal gland.

###### Description.

**Figure 4.**
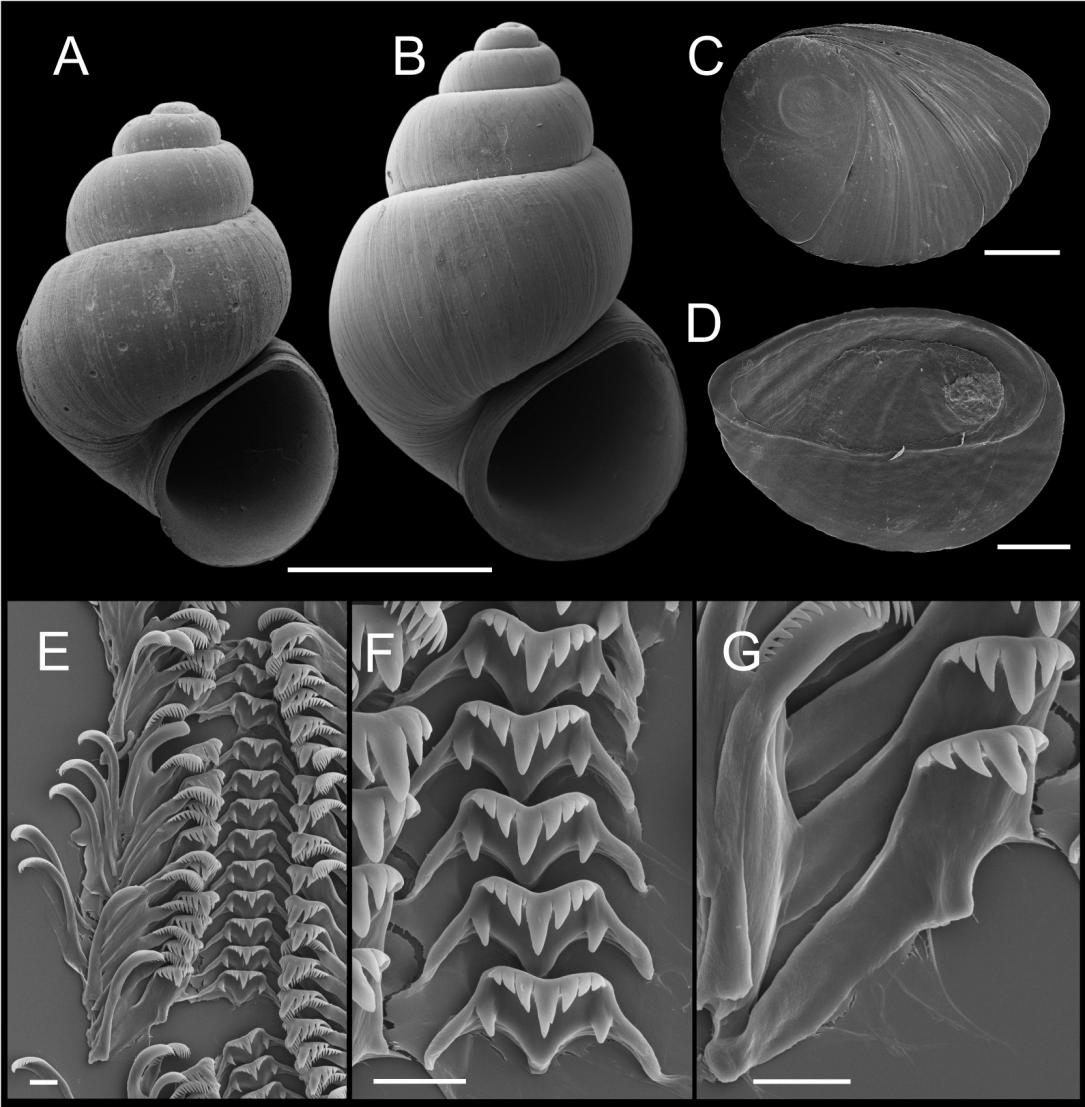
Shells, opercula and radula, *Pyrgulopsis lindae* sp. n. **A** Holotype, USNM 905250 **B** Shell, BellMNH 20821 **C, D** Opercula (outer, inner sides), BellMNH 20821 **E** Portion of radular ribbon, BellMNH 20821 **F** Central teeth, BellMNH 20821 **G** Lateral teeth, BellMNH 20821. Scale bars: **A–B** = 1.0 mm; **C–D** = 200 µm; **E–G** = 10 µm.

Shell (Fig. 4A–B, Table 3) ovate-conic, spire slightly longer than shell width in largest specimens, whorls 4.00–4.75. Teleoconch whorls medium convex, sometimes weakly shouldered. Aperture ovate, slightly angled above; parietal lip complete, nearly straight, narrowly disjunct, thin or slightly thickened; umbilicus absent or very small. Outer lip thin, orthocline. Teleoconch whorls sculptured with numerous irregular spiral striae.

**Table 3.** Shell parameters for *Pyrgulopsis lindae*. Measurements are in mm.

	WH	SH	SW	HBW	WBW	AH	AW	SW/SH	HBW/SH	AH/SH
Holotype, USNM 905258										
	4.25	2.46	1.61	1.79	1.43	1.06	0.94	0.65	0.73	0.43
BellMNH 20821 (n=17)										
Mean	4.56	2.83	1.83	2.08	1.57	1.21	1.10	0.65	0.74	0.43
S.D.	0.17	0.21	0.09	0.13	0.09	0.07	0.07	0.04	0.03	0.02
Range	4.25–4.75	2.65–3.33	1.63–1.95	1.87–2.34	1.40–1.76	1.09–1.31	1.01–1.18	0.59–0.68	0.69–0.77	0.39–0.47

Operculum (Fig. 4C–D) as for genus; muscle attachment margin thickened on inner side. Radula (Fig. 4E–G) as for genus; dorsal edge of central teeth concave, lateral cusps three–four, basal cusp one. Lateral teeth having two cusps on inner and three cusps on outer side. Inner marginal teeth with 15–20 cusps, outer marginal teeth with 22–28 cusps. Radula data are from BellMNH 20821.

Penis (Fig. 3A) medium-sized; filament darkly pigmented, medium length, narrow, tapering; lobe medium-sized, rectangular, slightly oblique; penial gland narrow, positioned along outer edge of filament basally; terminal gland narrow, curved, overlapping both dorsal and ventral sides of lobe; ventral gland small, narrow, curved, borne on short stalk near base of lobe. Penial data are from USNM 905259 (5 specimens), BellMNH 20821 (3 specimens).

###### Etymology.

This species is named for Linda Lee Crisostomo who provided invaluable field assistance and logistical support for this project. We propose that “San Domingo pyrg” be used as the common name for this species.

###### Distribution and habitat.

*Pyrgulopsis lindae* is known from three geographically proximate localities in the upper Calaveras and upper Tuolumne River basins. The type locality is a moderate-size stream of about one meter depth; specimens were found on emergent macrophytes near the banks. The second locality in San Domingo Valley is an old record (1929) based on dry shells. The place name for the third locality, “Salvada Gulch,” is no longer in use, but is shown on older maps (e.g., USGS Chinese Camp 15-minute quadrangle [1948]) as being located just to the east of Chinese Camp near the western edge of Don Pedro Reservoir. The geographic coordinates given on the original labels for the Salvada Gulch sample (BellMNH 2081) suggest that the collecting locality was the small stream just to the south of Shawmut Road.

###### Conservation status.

*Pyrgulopsis lindae* was found only rarely in San Domingo Creek in 2000; when re-visited in 2015 the creek consisted of a few pools separated by long, dry reaches; we were unable to sample these habitats as they were on fenced (private) land. The Salvada Gulch population has not been surveyed since it was first collected in 1966.

##### Pyrgulopsis ojaiensis

Taxon classificationAnimaliaLittorinimorphaHydrobiidae

Hershler, Liu, Babbitt, Kellogg & Howard sp. n.

http://zoobank.org/91F33C91-EEFB-4517-9255-740113055AF0

Figs 3B , 5

Pyrgulopsis stearnsiana .—Hershler and Liu 2010 (in part).

###### Types.

Holotype, SBMNH 74347, Sisar Creek, Santa Paula Canyon, 3.4 km up flow from Sulphur Springs, Ventura County, California, 34.43213°N, 119.12414°W, 1/7/1962, W. B. Miller. Paratypes, SBMNH 460496 (19 dry shells and ca. 100 alcohol preserved specimens), from same lot.

###### Referred material.

California. *Ventura County*: *USNM 905259, USNM 1287762, *ibid*., 6/23/2000, 6/26/2015.

###### Diagnosis.

A medium-sized congener (maximum shell height, 3.1 mm) having an ovate-conic shell. Distinguished from closely similar *Pyrgulopsis stearnsiana* and *Pyrgulopsis torrida* (described below) in having an oblique penial filament and larger penial lobe. Further differs from *Pyrgulopsis torrida* in having a longer penial filament and smaller terminal gland.

###### Description.

**Figure 5.**
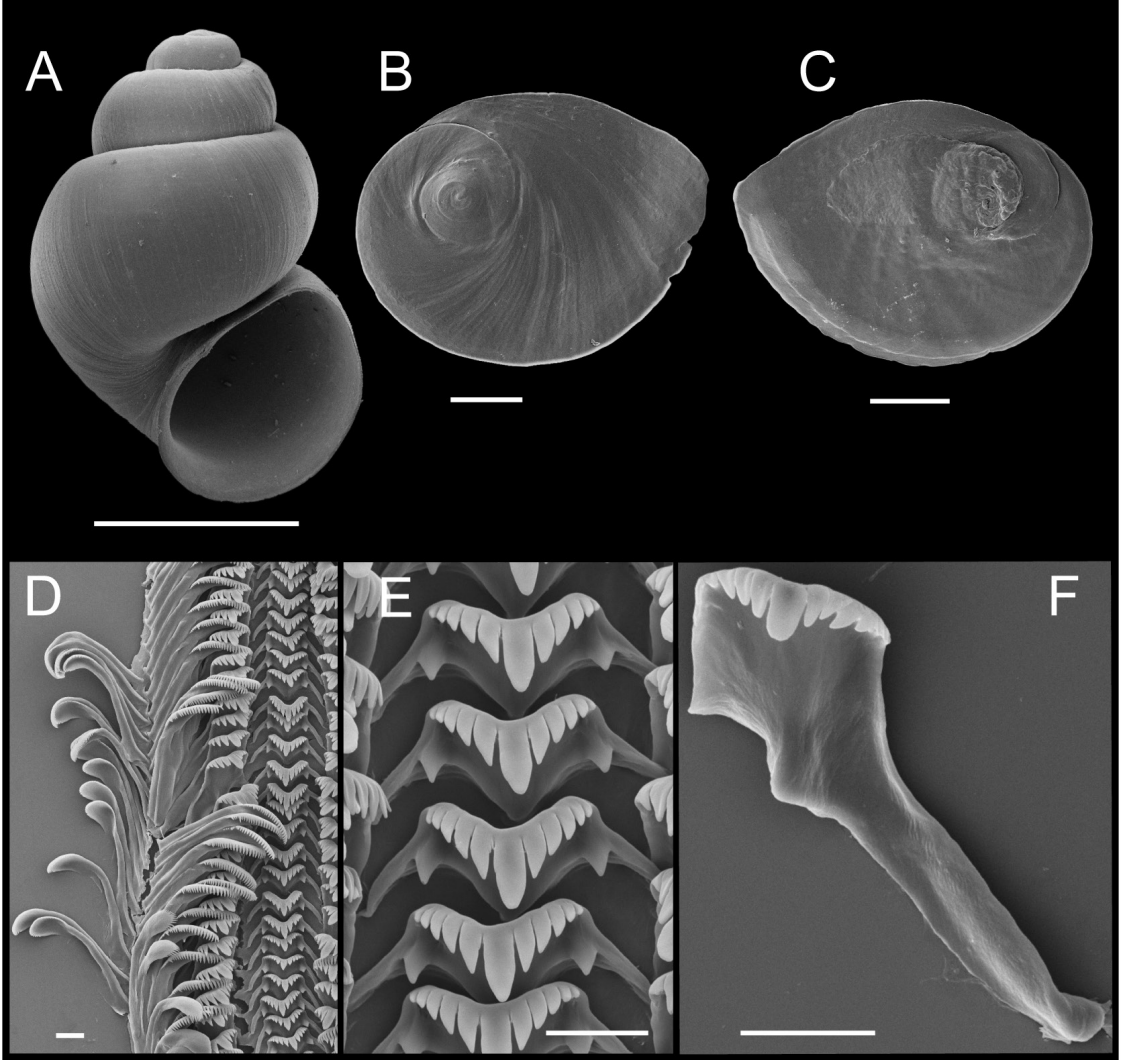
Shells, opercula and radula, *Pyrgulopsis ojaiensis* sp. n. **A** Holotype, SBMNH 74347 **B, C** Opercula (outer, inner sides), SBMNH 460496 **D** Portion of radular ribbon, SBMNH 460496 **E** Central teeth, SBMNH 460496 **F** Lateral teeth, SBMNH 460496. Scale bars: **A** =1.0 mm; **B–C** = 200 µm; **D–F** = 10 µm.

Shell (Fig. 5A, Table 4) ovate-conic, whorls 4.00–4.25. Teleoconch whorls medium convex, narrowly shouldered. Aperture ovate, slightly angled above; parietal lip complete, nearly straight, narrowly disjunct, last 0.25 whorl sometimes separated, thin or slightly thickened; umbilicus small. Outer lip thin, weakly prosocline or orthocline. Teleoconch smooth aside from collabral growth lines.

**Table 4.** Shell parameters for *Pyrgulopsis ojaiensis*. Measurements are in mm.

WH	SH	SW	HBW	WBW	AH	AW	SW/SH	HBW/SH	AH/SH
Holotype, SBMNH 70437									
4.00	2.51	1.72	1.95	1.50	1.17	1.07	0.69	0.78	0.47

Operculum (Fig. 5B–C) as for genus; inner side nearly smooth. Radula (Fig. 5D–F) as for genus; dorsal edge of central teeth concave, lateral cusps four–seven, basal cusp one. Lateral teeth having three–four cusps on inner and four–five cusps on outer side. Inner marginal teeth with 20–27 cusps, outer marginal teeth with 25–37 cusps. Radula data are from SBMNH 7437.

Penis (Fig. 3B) medium-sized (pigmentation unknown), filament medium length, narrow, oblique, tapering; lobe medium-sized, rectangular, oblique; terminal gland small, narrow, positioned along ventral edge of lobe. Penial data are from SBMNH 7437 (6 specimens).

###### Etymology.

The species name is a geographical epithet referring to Ojai Valley, the upper portion of which is drained by Sisar Creek. We propose “Sisar pyrg” as the common name for this species.

###### Distribution and habitat.

Endemic to the type locality; a small, spring-fed stream. Snails were found on small stones and pieces of wood.

###### Conservation status.

*Pyrgulopsis ojaiensis* was found in moderate abundance in Sisar Creek both in 2000 and 2015. This creek runs alongside a frequently used road (between Ojai and Santa Paula) in a populated area and has been considerably impacted by anthropogenic activities.

##### Pyrgulopsis torrida

Taxon classificationAnimaliaLittorinimorphaHydrobiidae

Hershler, Liu, Babbitt, Kellogg & Howard sp. n.

http://zoobank.org/2FBB4B8B-32C2-4308-AB78-C8454A1B8ED1

Figs 3C , 6

Pyrgulopsis stearnsiana .—Hershler and Liu 2010 (in part).

###### Types.

Holotype, SBMNH 74238, Little Sycamore Canyon, creek 3.2 km up flow from Hwy 1, Ventura County, California, 34.07509°N, 118.95508°W, 11/11/1961, W. B. Miller. Paratypes, SBMNH 460492 (ca. 200 dried shells), from same lot.

###### Referred material.

California. *Ventura County*: SBMNH 74236, *USNM 1120443, *ibid*, 9/9/1956, 10/21/2008, respectively.

###### Diagnosis.

A medium-sized congener (maximum shell height, 2.8 mm) having an ovate-conic shell. Distinguished from *Pyrgulopsis stearnsiana* by its shorter penial filament and larger terminal gland.

###### Description.

**Figure 6.**
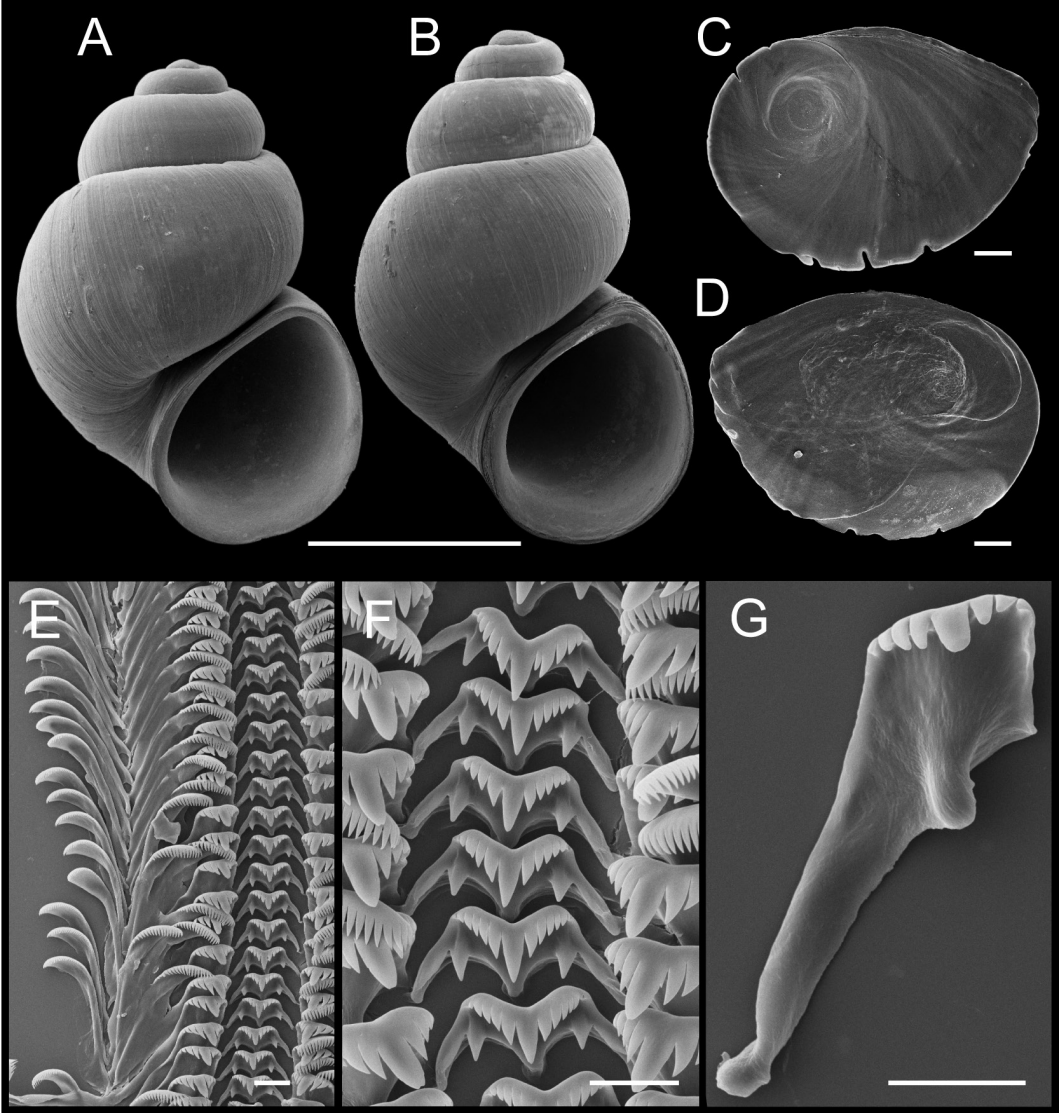
Shells, opercula and radula, *Pyrgulopsis torrida* sp. n. **A** Holotype, SBMNH 74238 **B** Shell, SBMNH 460492 **C, D** Opercula (outer, inner sides), SBMNH 460492 **E** Portion of radular ribbon, SBMNH 460492 **F** Central teeth, SBMNH 460492 **G** Lateral teeth, SBMNH 460492. Scale bars: **A–B**=1.0 mm; **C–D**=100 µm; **E–G**=10 µm.

Shell (Fig. 6A–B, Table 5) ovate-conic, whorls 4.00. Teleoconch whorls medium convex, narrowly shouldered. Aperture ovate, slightly angled above; parietal lip complete, nearly straight, narrowly adnate adapically or slightly disjunct, thin or slightly thickened; umbilicus small. Outer lip thin, orthocline. Teleoconch smooth or sculptured with weak spiral striae.

**Table 5.** Shell parameters for *Pyrgulopsis torrida*. Measurements are in mm.

	WH	SH	SW	HBW	WBW	AH	AW	SW/SH	HBW/SH	AH/SH
Holotype, SBMNH 74238										
	4.00	2.511	1.72	1.96	1.45	1.21	1.04	0.68	0.79	0.48
SBMNH 460492 (n=17)										
Mean	3.94	2.53	1.71	1.98	1.44	1.19	1.06	0.68	0.78	0.47
S.D.	0.17	0.10	0.10	0.07	0.07	0.06	0.05	0.04	0.03	0.03
Range	3.75–4.25	2.33–2.69	1.56–1.92	1.87–2.13	1.34–1.59	1.07–1.29	0.97–1.16	0.61–0.73	0.73–0.82	0.42–0.51

Operculum (Fig. 6C–D) as for genus; portion of attachment scar margin slightly thickened on inner side. Radula (Fig. 6E–G) as for genus; dorsal edge of central teeth concave, lateral cusps three–six, basal cusps one to (rarely) two. Lateral teeth having two–four cusps on inner and three–five cusps on outer side. Inner marginal teeth with 19–24 cusps, outer marginal teeth with 21–27 cusps. Radula data are from SBMNH 460492.

Penis (Fig. 3C) small, filament weakly pigmented or pale, filament short, narrow, horizontal, weakly tapering; lobe small, rectangular, horizontal; terminal gland fairly large, narrow, overlapping dorsal and ventral edges of lobe. Penial data are from USNM 1120443 (2 specimens).

###### Etymology.

The species name is an adjective derived from the New Latin *torridus*, meaning dry or parched, and refers to the recent desiccation of the stream in Little Sycamore Canyon. We propose “Little Sycamore pyrg” as the common name for this species.

###### Distribution.

Endemic to the type locality, a small, shallow stream which runs for about 1.6 km. Snails were collected from the mud bottoms of a series of small puddle-like pools along the middle section of the stream.

###### Conservation status.

*Pyrgulopsis torrida* was found only rarely in the Little Sycamore Canyon creek in 2000. The entirely length of the canyon was dry when re-visited in 2015, suggesting that this population may now be extirpated.

## Discussion

### Taxonomic status of *Pyrgulopsis stearnsiana*

**Figure 7.**
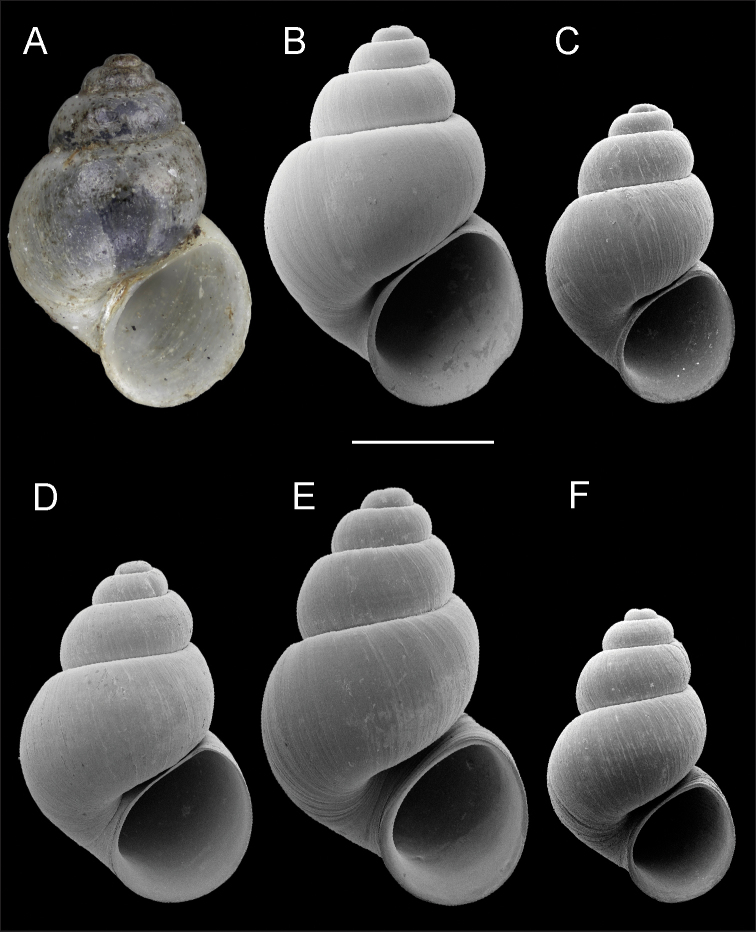
Shells, *Pyrgulopsis stearnsiana*. **A** Lectotype, ANSP 27961 **B** USNM 1291731 **C** USNM 894756 **D** USNM 1287760 **E** USNM 1287759 **F** USNM 1252041. Scale bar: 1.0 mm.

As is often the case with animal species (Funk and Omland 2003; Ross 2014), *Pyrgulopsis stearnsiana*, as newly circumscribed herein, was resolved as a paraphyletic assemblage based on mtDNA evidence (Fig. 2; also see Liu et al. 2003, figs 2–3; Hershler and Liu 2010, fig. 2). The two congeners that are nested within the *Pyrgulopsis stearnsiana* clade—*Pyrgulopsis diablensis* and *Pyrgulopsis giulianii*—are somewhat distinct genetically (differing from *Pyrgulopsis stearnsiana* *sensu stricto* by 1.5% and 1.9% for COI and 2.0% and 2.3% for NDI; Tables 1–2) and are further differentiated by their penes: *Pyrgulopsis diablensis* has a small penial gland (Hershler 1995, fig. 3B; observed in 29/30 specimens from USNM 883791), and *Pyrgulopsis giulianii* has both a ventral gland (30/30 specimens, USNM 874141) and a gland on the dorsal penis proximal to the base of the filament (28/30 specimens, USNM 874141) (Hershler and Pratt 1990, fig. 3). The distinction between *Pyrgulopsis stearnsiana* and *Pyrgulopsis diablensis* is somewhat blurred as a small penial gland (diagnostic of the latter) was detected at a very low frequency in the *Pyrgulopsis stearnsiana* material that we examined during the course of this study (12/156 specimens from seven populations; BellMNH 20811, BellMNH 20814, BellMNH 20932, USNM 874181, USNM 905251, USNM 1152039, USNM 1252041). Nonetheless, we do not see a compelling basis for treating the entire *stearnsiana* clade as a single species (and thus “avoiding” paraphyly), especially given the very clear morphological distinction between *Pyrgulopsis stearnsiana* and *Pyrgulopsis giulianii*. Our molecular data suggest that a more appropriate action may be to further split *Pyrgulopsis stearnsiana* taxonomically. The sequence divergence within *Pyrgulopsis stearnsiana* was fairly large—2.0 +/- 0.3% for COI (Table 1) and 2.4 +/- 0.4% for NDI (Table 2)—and five small subclades of these snails were well supported. Furthermore, *Pyrgulopsis stearnsiana* is morphologically variable, especially in shell size and shape (Fig. 7A–F), and shape of the penial filament and terminal gland (Fig. 3D–F). However, we do not have a sufficiently robust dataset at this time to confidently tease apart this cryptic diversity, although we anticipate being able to do so when data from a rapidly evolving molecular marker (such as microsatellites) become available. Thus, for the time being, we recognize only three species in the *Pyrgulopsis stearnsiana* clade (*Pyrgulopsis diablensis*, *Pyrgulopsis giulianii*, *Pyrgulopsis stearnsiana*) while acknowledging that the paraphyly of *Pyrgulopsis stearnsiana* is probably an artifact of incompletely resolved taxonomy.

### Conservation considerations

Springsnails are a current focus of conservation attention in many parts of the West owing to the threats posed by groundwater pumping, surface water diversions, and other anthropogenic activities (Hershler et al. 2014b). All three of the new species described herein are narrowly distributed (note that the precise limits of these geographic ranges are uncertain) and for this reason alone should be placed on conservation watch lists. Although the three species were extant in 2000, only one of them (*Pyrgulopsis ojaiensis*) was found in 2015. The habitats of *Pyrgulopsis lindae* and *Pyrgulopsis torrida* were severely impacted by the recent (2012-2015), extreme California drought (Robeson 2015) and it appears likely that the single known population of the latter species has been extirpated. Field surveys are needed to determine (1) whether *Pyrgulopsis torrida* may have re-populated the creek in Little Sycamore Canyon and whether there are other, previously unknown populations in the southern California coastal drainage; and (2) to similarly assess whether *Pyrgulopsis lindae* is extant in San Domingo Creek and Salvada Gulch. The remaining habitats of the three species may require protective measures to ensure their persistence; we note in this context that all of the known localities for these snails are on private land.

During the course of our fieldwork we also found that quite a few populations of *Pyrgulopsis stearnsiana* have recently (post-1960) become extirpated, including, for example, those in Palo Seco Creek, San Leandro Creek, and Russellman Park Spring in the San Francisco Bay area. In most of these cases the previously inhabited spring or stream is now dry. Most of the extant populations of *Pyrgulopsis stearnsiana* live in small water bodies (springs or streams) that have been variously impacted by anthropogenic activities (e.g., flow diversions, recreational use, livestock grazing). *Pyrgulopsis stearnsiana* is currently ranked as imperiled (G2) by NatureServe (2015), threatened by the American Fisheries Society (Johnson et al. 2013), and as a species of Least Concern by the IUCN (Cordeiro and Perez 2011). We recommend that the conservation rankings of *Pyrgulopsis stearnsiana* be updated to reflect the recent spate of population extirpations and the highly modified condition of the remaining habitats of this snail.

## Supplementary Material

XML Treatment for Pyrgulopsis lindae

XML Treatment for Pyrgulopsis ojaiensis

XML Treatment for Pyrgulopsis torrida

## Supplementary materials

Supplementary material 1 Specimen codes, number of sequenced specimens, locality details, and GenBank accession numbers Data type: species data This dataset is made available under the Open Database License (http://opendatacommons.org/licenses/odbl/1.0/). The Open Database License (ODbL) is a license agreement intended to allow users to freely share, modify, and use this Dataset while maintaining this same freedom for others, provided that the original source and author(s) are credited.Robert Hershler, Hsiu-Ping Liu, Caitlin Babbitt, Michael G. Kellogg, Jeanette K. Howard

Supplementary material 2 Specimens of *Pyrgulopsis stearnsiana* sensu stricto that were examined as part of this study Data type: specimens data Explanation note: All material is from California. This dataset is made available under the Open Database License (http://opendatacommons.org/licenses/odbl/1.0/). The Open Database License (ODbL) is a license agreement intended to allow users to freely share, modify, and use this Dataset while maintaining this same freedom for others, provided that the original source and author(s) are credited.Robert Hershler, Hsiu-Ping Liu, Caitlin Babbitt, Michael G. Kellogg, Jeanette K. Howard
